# Colpocleisis for recurrent pelvic organ prolapse after tension-free vaginal mesh repair: a case report

**DOI:** 10.1093/jscr/rjaf537

**Published:** 2025-07-14

**Authors:** Akihiko Misawa, Eizo Kimura, Miho Inoue, Shion Mitsuya, Makoto Osaka, Yasunori Sato, Kiyono Osanai, Atsushi Suzuki

**Affiliations:** Department of Gynecology, Asoka Hospital, 1-18-1 sumiyoshi, Koyo-ku, Tokyo 135-0002, Japan; Department of Obstetrics and Gynecology, Kyorin Suginami Hospital, 2-25-1 wada, Suginami-ku, Tokyo 166-0012, Japan; Department of Gynecology, Higirigaoka Hospital, 2-69-6 renkoji, tama-city, Tokyo 206-0021, Japan; Department of Obstetrics and Gynecology, Kyorin Suginami Hospital, 2-25-1 wada, Suginami-ku, Tokyo 166-0012, Japan; Department of Obstetrics and Gynecology, Kyorin Suginami Hospital, 2-25-1 wada, Suginami-ku, Tokyo 166-0012, Japan; Department of Obstetrics and Gynecology, Kyorin Suginami Hospital, 2-25-1 wada, Suginami-ku, Tokyo 166-0012, Japan; Department of Obstetrics and Gynecology, Kyorin Suginami Hospital, 2-25-1 wada, Suginami-ku, Tokyo 166-0012, Japan; Department of Obstetrics and Gynecology, Kyorin Suginami Hospital, 2-25-1 wada, Suginami-ku, Tokyo 166-0012, Japan; Department of Obstetrics and Gynecology, Kyorin Suginami Hospital, 2-25-1 wada, Suginami-ku, Tokyo 166-0012, Japan

**Keywords:** tension-free vaginal mesh (TVM), recurrent prolapse, LeFort colpocleisis, native tissue repair (NTR)

## Abstract

Tension-free vaginal mesh repair surgery (TVM) has decreased due to complications related to mesh use and the lack of superiority compared to native tissue repair (NTR). In japan it is still performed as a treatment for pelvic organ prolapse (POP). However, recurrent prolapse after TVM does occur, necessitating appropriate management. The patient was underwent TVM-A surgery at another hospital 12 years ago for cystocele. POP-Q stage II rectocele was observed, and the LeFort colpocleisis was performed. Although TVM surgery is effective via the vaginal approach, recurrent posterior vaginal wall prolapse may occur over time, as seen in this case, even in patients who had single-mesh procedures for anterior vaginal wall repair. When conservative treatment is difficult, vaginal closure surgery, a traditional approach, can be safely performed.

## Introduction

Tension-free vaginal mesh (TVM) surgery was introduced in 2004 as a standardized procedure to address the 20%–30% recurrence rate associated with traditional native tissue repair (NTR) procedures [1]. Since then, TVM has rapidly gained popularity. However, there has been an increasing number of lawsuits due to chronic pain, infections, mesh migration or damage, tissue damage caused by the mesh, dyspareunia, and other quality-of-life issues. Concerns about its safety were raised in the United States, and TVM was subsequently banned after it failed to demonstrate superiority over NTR [2, 3]. TVM is no longer thought of as inferior to NATR in surgical outcomes and failure rates in the United States after the eSUPER and ASPIRE trials although it is still banned. Other countries, including Canada, the UK, Australia, New Zealand, and France, have also banned or restricted the use of mesh surgeries. In Japan, polytetrafluoroethylene mesh is still used, although the number of cases has decreased. While the recurrence rate and the need for reoperation after TVM are lower compared to NTR, a certain number of cases still require intervention [3].

In this report, we present a case of a patient who presented with a sensation of vaginal prolapse 12 years after undergoing TVM-A surgery at another hospital, was diagnosed with rectocele, and underwent the colpocleisis.

## Case report

An 81-year-old woman, gravida 2, para 2 (both normal vaginal deliveries), underwent a total abdominal hysterectomy approximately at 40 years old for uterine fibroids. Twelve years ago, she had TVM-anterior (TVM-A) surgery at another hospital for cystocele. About one month before presenting to her previous physician, she experienced worsening post vaginal wall prolapse. Although a non-invasive pessary was attempted, she experienced significant discomfort and opted for surgery, leading to her referral to our hospital. At the initial consultation, a rectocele was noted, with the posterior vaginal wall prolapsing beyond the vaginal opening. No cystocele was observed due to the previous TVM-A surgery. Ultrasonography revealed no other abnormalities, such as ovarian enlargement. We diagnosed rectal prolapse in Stage II. She wanted the least invasive surgery possible. Given her history of total hysterectomy and TVM-A surgery, as well as her age, she opted for the colpocleisis, which is less invasive.

**Figure 1:**
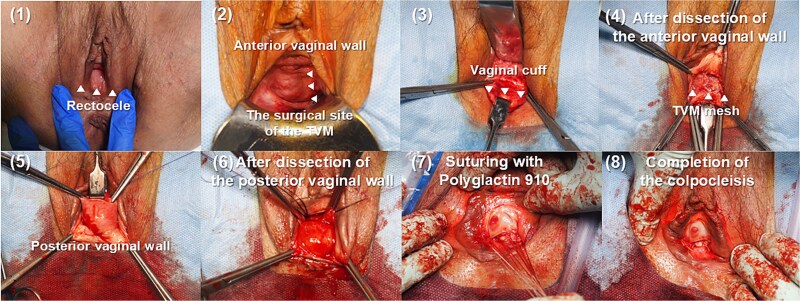
Presentation of the colpocleisis procedure. The figure illustrates the initial dissection of the anterior and posterior vaginal walls, the subsequent suturing using polyglactin 910, and the final closure of the vaginal canal.

Surgery: The vaginal cuff, following the total hysterectomy, was identified and retracted. The anterior mesh was effective, as retraction of the vaginal cuff caused only minimal prolapse of the anterior wall. The anterior vaginal wall was dissected in a trapezoidal shape from ~1 cm from the vaginal cuff to ~1 cm from the urethral opening. During the anterior wall dissection, part of the mesh sutures were observed. The posterior vaginal wall was dissected from ~1 cm from the vaginal cuff to the hymen, and the colpocleisis was performed by suturing the dissection plane. (Fig. 1) The operation took 64 minutes, and blood loss was 7 g. The patient was discharged on the 8th day without any complications such as infections. About 18 months postoperatively, there were no signs of recurrence or infection, and the patient’s recovery was uneventful.

## Discussion

Postoperative complications of TVM include wound infections, mesh erosion or exposure, voiding dysfunction, urinary incontinence, pain, and recurrence. Due to the decrease in quality of life associated with these complications, lawsuits have increased. In the U.S. Food and Drug Administration's Coloplast Transvaginal Mesh for pelvic organ prolapse (POP) 522 study, non-inferiority in terms of safety was demonstrated. However, TVM mesh failed to establish superiority over NTR in terms of recurrence rates, leading to a ban on TVM mesh in the United States [4]. Additionally, although TVM has a lower reoperation rate for recurrence compared to NTR, the reoperation rate due to complications is higher for TVM [3, 5]. Consequently, the risk–benefit profile suggests that the usefulness of transvaginal mesh in primary surgeries is limited.In contrast, the success rate of Lefort colpocleisis and total vaginal prolapse surgery, which have been performed for a long time, >90%, with high patient satisfaction [6].

With the recent development of laparoscopic surgery and robot-assisted surgery, there are various options for surgical procedures. Surgical treatments for recurrent cases include the laparoscopic pectopexy and paravaginal repair [7], the laparoscopic sacral colpopexy [8, 9], and the colpocleisis [10]. In elderly women, such as in the present case, LeFort colpocleisis has been shown to be a feasible and safe option for recurrent POP [10].

In this case, considering the recurrence after TVM and the patient's advanced age of 81, we opted for a surgical method with a short duration and fewer complications. Additionally, since the patient did not want another surgery involving synthetic materials, the colpocleisis was chosen. The short-term prognosis and patient satisfaction have been favorable. LeFort colpocleisis is a classic surgical procedure. However, it is less invasive, takes less time, and patients are satisfied with the results.

## Conclusion

While TVM surgery is effective for transvaginal procedures, cases like the present one, involving single-mesh repair of the anterior vaginal wall, may still result in recurrence of posterior vaginal wall prolapse over time, requiring further surgical intervention. When conservative treatment is difficult, the traditional colpocleisis can be performed safely and effectively.

## References

[1] Debodinance P, Berrocal J, Clavé H, et al. Évolution des idées Sur le traitement chirurgical des prolapsus génitaux: naissance de la technique TVM. J Gynecol Obstet Biol Reprod (Paris) 2004;33:577–88. 15550876 10.1016/s0368-2315(04)96598-2

[2] Food and Drug Administration . Urogynecologic surgical mesh: Update on the safety and effectiveness of transvaginal placement for pelvic organ prolapse. Available from: https://www.fda.gov/media/81123/download (11 Oct 2024, date last accessed).

[3] Yeung E, Baessler K, Christmann-Schmid C, et al. Transvaginal mesh or grafts or native tissue repair for vaginal prolapse. Cochrane Database Syst Rev 2024;2024:CD012079. 10.1002/14651858.CD012079.pub2 PMC10936147 38477494

[4] Food and Drug Administration . Coloplast transvaginal mesh for pelvic organ prolapse (POP) 522 study. Available from: https://www.accessdata.fda.gov/scripts/cdrh/cfdocs/cfPMA/pss.cfm?t_id=292&c_id=739 (16 June 2025, date last accessed).

[5] Capobianco G, Sechi I, Muresu N, et al. Native tissue repair versus transvaginal mesh interventions for the treatment of anterior vaginal prolapse: systematic review and meta-analysis. Maturitas 2022;165:104–12. 10.1016/j.maturitas.2022.07.013 35963180

[6] Buchsbaum GM, Lee TG. Vaginal obliterative procedures for pelvic organ prolapse: a systematic review. Obstet Gynecol Surv 2017;72:175–83. 28304415 10.1097/OGX.0000000000000406

[7] Bakir MS, Türkay U, Bakır VL, et al. Laparoscopic pectopexy and paravaginal repair after failed recurrent pelvic organ prolapse surgery. Gynecol Minim Invasive Ther 2020;9:42–44. 10.4103/GMIT.GMIT_101_18 PMC7008650 32090014

[8] Schmid C, Brucker SY, Hadaschik EN, et al. Laparoscopic sacrocolpopexy for recurrent pelvic organ prolapse after failed transvaginal polypropylene mesh surgery. Int Urogynecol J 2013;24:763–7. 10.1007/s00192-012-1926-5 22976531

[9] Studer AM, Faehnle-Schiegg I, Frey J, et al. Recurrent pelvic organ prolapse after sacrocolpopexy – a surgical challenge. J Clin Med 2024;13:1613. 10.3390/jcm13061613 38541839 PMC10970834

[10] Wang X, Hu C, Chen Y, et al. LeFort colpocleisis for recurrent pelvic organ prolapse. Int Urogynecol J 2020;31:381–4. 10.1007/s00192-019-03969-y 31069411

